# Quantifying the burden of vampire bat rabies in Peruvian livestock

**DOI:** 10.1371/journal.pntd.0006105

**Published:** 2017-12-21

**Authors:** Julio A. Benavides, Elizabeth Rojas Paniagua, Katie Hampson, William Valderrama, Daniel G. Streicker

**Affiliations:** 1 Institute of Biodiversity, Animal Health and Comparative Medicine, University of Glasgow, Graham Kerr Building, Glasgow, Scotland, United Kingdom; 2 Association for the Conservation and Development of Natural Resources, Lima, Peru; 3 MRC-University of Glasgow Centre for Virus Research, Sir Henry Wellcome Building, Glasgow, Scotland, United Kingdom; Wistar Institute, UNITED STATES

## Abstract

**Background:**

Knowledge of infectious disease burden is necessary to appropriately allocate resources for prevention and control. In Latin America, rabies is among the most important zoonoses for human health and agriculture, but the burden of disease attributed to its main reservoir, the common vampire bat (*Desmodus rotundus*), remains uncertain.

**Methodology/Principal findings:**

We used questionnaires to quantify under-reporting of livestock deaths across 40 agricultural communities with differing access to health resources and epidemiological histories of vampire bat rabies (VBR) in the regions of Apurimac, Ayacucho and Cusco in southern Peru. Farmers who believed VBR was absent from their communities were one third as likely to report livestock deaths from disease as those who believed VBR was present, and under-reporting increased with distance from reporting offices. Using generalized mixed-effect models that captured spatial autocorrelation in reporting, we project 4.6 (95% CI: 4.4–8.2) rabies cases per reported case and identify geographic areas with potentially greater VBR burden than indicated by official reports. Spatially-corrected models estimate 505–724 cattle deaths from VBR in our study area during 2014 (421–444 deaths/100,000 cattle), costing US$121,797–171,992. Cost benefit analysis favoured vaccinating all cattle over the current practice of partial vaccination or halting vaccination all together.

**Conclusions:**

Our study represents the first estimate of the burden of VBR in Latin America to incorporate data on reporting rates. We confirm the long-suspected cost of VBR to small-scale farmers and show that vaccinating livestock is a cost-effective solution to mitigate the burden of VBR. More generally, results highlight that ignoring geographic variation in access to health resources can bias estimates of disease burden and risk.

## Author summary

The number of cases and monetary cost of a disease guides how resources for prevention and control are allocated. In Latin America, rabies transmitted by vampire bats is one of the most recognized zoonoses affecting humans and livestock, but its burden on lives and livelihoods has been difficult to calculate because the percentage of outbreaks that are not reported to surveillance systems is unknown. Here, using surveys to calculate farmers’ tendencies to report livestock deaths, we estimate that over 500 cattle died of rabies in southern Peru in 2014, a loss of approximately US$170,000 or over 700 months of local income. Our results also show that the perceived risk of rabies strongly affected reporting of cattle mortality and vaccination coverage, suggesting that campaigns to increase awareness could reduce the burden of rabies.

## Introduction

Knowledge of the number of cases and associated economic losses from infectious diseases (“disease burden”) is crucial to allocate resources for prevention and control appropriately. Estimating disease burden is a priority for the control of neglected zoonoses [1] but is challenging, particularly in low- and middle-income countries (LMICs) where passive surveillance systems face chronic but typically unquantified under-reporting of cases. This can create large discrepancies between the officially reported and the actual disease burden [2]. Community-based studies (CBS) can complement passive surveillance systems by asking communities directly about disease events [3–5]. CBS are routinely used to quantify a range of parameters needed to estimate disease burden, including under-reporting and the costs of outbreaks. CBS can also improve estimates of parameters crucial for disease prevention and control, such as vaccination uptake [6, 7]. Associations between reporting and vaccination and more widely measured variables such as socio-economic status are commonly used to extrapolate disease burden and vaccine uptake across larger geographic areas [8].

In Latin America, rabies is considered among the most important zoonoses for human and animal health [9]. The common vampire bat (*Desmodus rotundus*) is the principle reservoir throughout the region. Main activities for prevention and control include culling bats using poison and vaccination of humans and livestock [10]. The burden of vampire bat-transmitted rabies (VBR) on human lives and livelihoods is largely anecdotal [11, 12]. Livestock losses across Latin America were estimated as roughly US$100 million annually in the 1960s and US$50 million annually in the 1990s [11], including US$15 million in Brazil alone [13, 14]. However, to our knowledge these estimates were based on assumed rates of under-reporting and rabies prevalence, making quantitative valuation of the benefits of interventions difficult. These uncertainties contribute to neglect that ultimately increases the burden of the disease [15–17].

In Peru, geographic expansions of VBR have raised serious concerns for agriculture and public health [15, 18]. Like most Latin American countries, Peru maintains passive surveillance for rabies and other infectious diseases of livestock that rely on community reporting of suspected outbreaks. However, neither the average rate of under-reporting nor the extent of variation in under-reporting across communities is known. One important source of variation in disease reporting and in preventative behaviours such as vaccination is the degree of access to health resources [19, 20]. This is particularly important in LMICs such as Peru, where poor transport and accessibility in rural areas limits the use of health resources [21, 22]. Although geographic isolation is widely acknowledged as an important factor influencing reporting and vaccination uptake [19, 20, 23–25], it is not typically incorporated in studies estimating disease burden. Independently of spatial effects, reporting and prevention practices may increase with socio-economic status [20, 26] because those with higher incomes are better able to access health resources and to pay for vaccines. Reporting and vaccination may also increase with perceptions of heightened disease risk [27, 28], greater trust in the health authorities [19, 29] and better knowledge of veterinary services [25]. Finally, environmental features underlying risk may be important. For example in vampire bat rabies, elevation may influence both the presence of vampire bats and reporting practices [30].

The purpose of this study was to estimate under-reporting rates using surveys and use these rates to project the actual number of VBR cases from reports to the official passive surveillance system. We focused on a region in the southern Peruvian Andes where VBR remains poorly controlled [15]. Specifically, by linking questionnaires on infectious diseases of livestock in 40 communities with passive surveillance data on 11 years of VBR outbreaks, we (i) quantify under-reporting and vaccination rates for VBR; (ii) identify predictors of the observed spatiotemporal variation in disease reporting and vaccination; (iii) estimate the burden of VBR accounting for this spatial variation in reporting; (iv) visualize how reporting biases may alter perceptions of geographic hot spots of rabies burden that might affect control decisions and (v) compare the costs and benefits of alternative vaccination scenarios relative to current vaccination coverage.

## Methods

### Ethics statement

All participants were read a consent form (including study objectives, risks and benefits for participants, confidentiality and that participation was voluntary) and received clarification if requested before signing. Participants also received a leaflet explaining the project, a copy of their written consent and contact information to request study results, and a pair of cattle identification tags as reimbursement for their time after the interview. The study was approved by the Ethics Committee of the College of Veterinary Science, University of Glasgow.

### Study area

**Fig 1:**
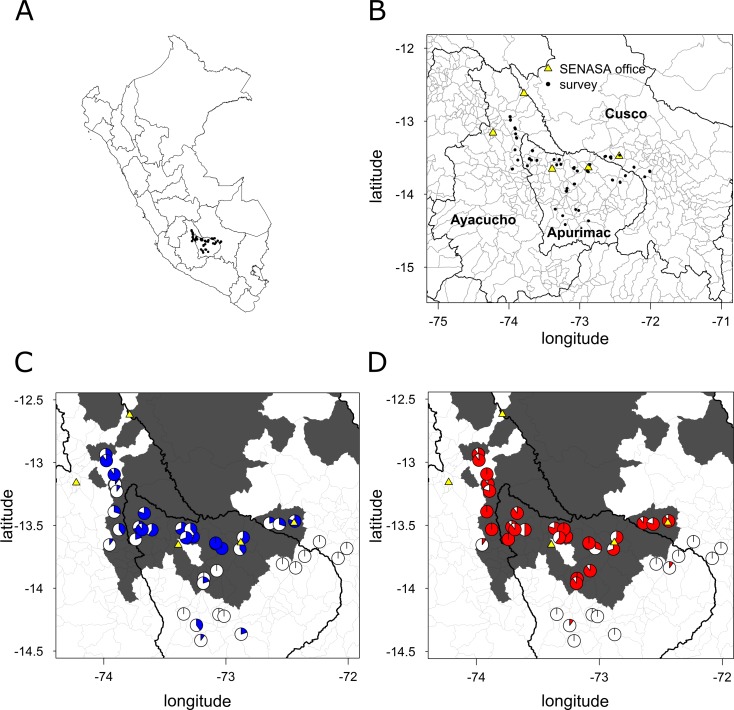
Reporting and vaccination rates per community across the study area. (A) Map of Peru with the location of surveys (B) Zoomed map showing surveys in the Ayacucho, Apurimac and Cusco regions. Yellow triangles show the location of the SENASA offices where cases are reported (C) Blue pie-charts show the percentage of farmers reporting cattle mortality from a suspected infectious disease in each community (N = 10 per community). Districts that previously reported one or more laboratory-confirmed cases of VBR are coloured grey. (D) Red pie-charts show the percentage of farmers vaccinating their cattle against rabies. Country, region and district maps were obtained from the GADM (http://www.gadm.org//) database using the *getData* function from the *raster* package of R.

Questionnaires were conducted during 2015 in the regions of Apurimac, Ayacucho and Cusco, which account for almost 70% of reported rabies cases in Peru and have been affected by VBR for at least three decades [10, 15, 18, 30]. Altogether, these regions have a human population of approximately 2.5 million [31] and a cattle population of approximately 1.1 million heads in around 185,000 farms according to the 2012 Agricultural Census of Peru (CENAGRO IV). Communities were chosen using a stratified random sampling procedure. First, we divided districts with (N = 280) and without (N = 120) a confirmed report of VBR to the National Service of Animal Health (SENASA, Fig 1). From each set of districts, we selected communities that were accessible by public transport and that were located between 0 and 100km from a SENASA office. From each community, we obtained a list of households that kept livestock from the local community leaders or farmers’ representatives and randomly selected 10 households for inclusion.

### Questionnaires

Questionnaires included 53 questions covering disease knowledge, reporting practices, prevention practices, knowledge of vampire bats, as well as information about socio-economic status. Interviews lasted around 1 hour and were performed in Quechua or Spanish by E.P. and J.B. Questionnaires were first validated on a small number of farmers (N = 10) to test the clarity of questions and farmers’ comprehension, and revised where necessary. Community leaders were informed of our study objectives. Data used in this study are available in S1 Table.

### Factors associated with livestock disease reporting and vaccination against VBR

We analysed (i) the factors associated with general disease reporting (the death of a sick cow regardless of clinical signs) and (ii) the factors associated with vaccinating cattle against VBR (a rabies-specific preventative action). We included the following factors which were calculated from questionnaires (S2 Table): perception of risk (i.e., farmer believes the disease has been present in local livestock) for six diseases that currently or historically affected livestock in the area (mainly VBR but also Clostridiosis by *Clostridium chauvoei*, brucellosis, swine vesicular disease, bovine tuberculosis and foot-and-mouth disease (FMD)), household socio-economic status (SES), the participant’s gender, age, knowledge of the role of SENASA and confidence in whether SENASA would investigate a reported livestock death, and the number of animals present. An additional factor, the distance from each farm to the closest SENASA reporting office (also the main supplier of vaccines) was calculated using a modification of the least-cost path distance described in Benavides *et al*. [15]. Specifically, we used the road map of the area and applied a conductance of 1 to all roads, while assuming that paths outside of roads were twice (and up to five times) as costly to follow. Results were unchanged at higher levels of resistance for non-road travel. We described differences in the SES of farmers using a Principal Components Analysis (PCA) that included 13 variables related to SES [32] (see S2 Table for a list of variables). The first two principal components accounted for 20% and 12% of the total variation respectively and were used as indicators of SES in later analyses.

The binary nature of our response variables (i.e. report or not and vaccinate or not) and the possibility of community-level differences in reporting that were not captured by our explanatory variables required using generalized linear mixed models with binomial errors (i.e. logistic regression). Furthermore, since surveys were spread across a large geographic area, our analysis needed to account for potential spatial autocorrelation, which occurs when values of variables sampled at close locations are more similar than those sampled far from each other [33]. Thus, following Dormann *et al*. [33], analyses used generalized quasi-likelihood linear mixed models (glmmPQL) to include both spatial autocorrelation and the identity of the community as random effects. All models were built using the glmmPQL function of the MASS package in R 3.2.1 [34–35]. The significance of spatial autocorrelation for the raw data and the residuals of each model were tested using the Moran’s I test [36] in the ape package of R [37].

### Estimation of the multiplication factor and burden of vampire bat rabies

**Fig 2:**
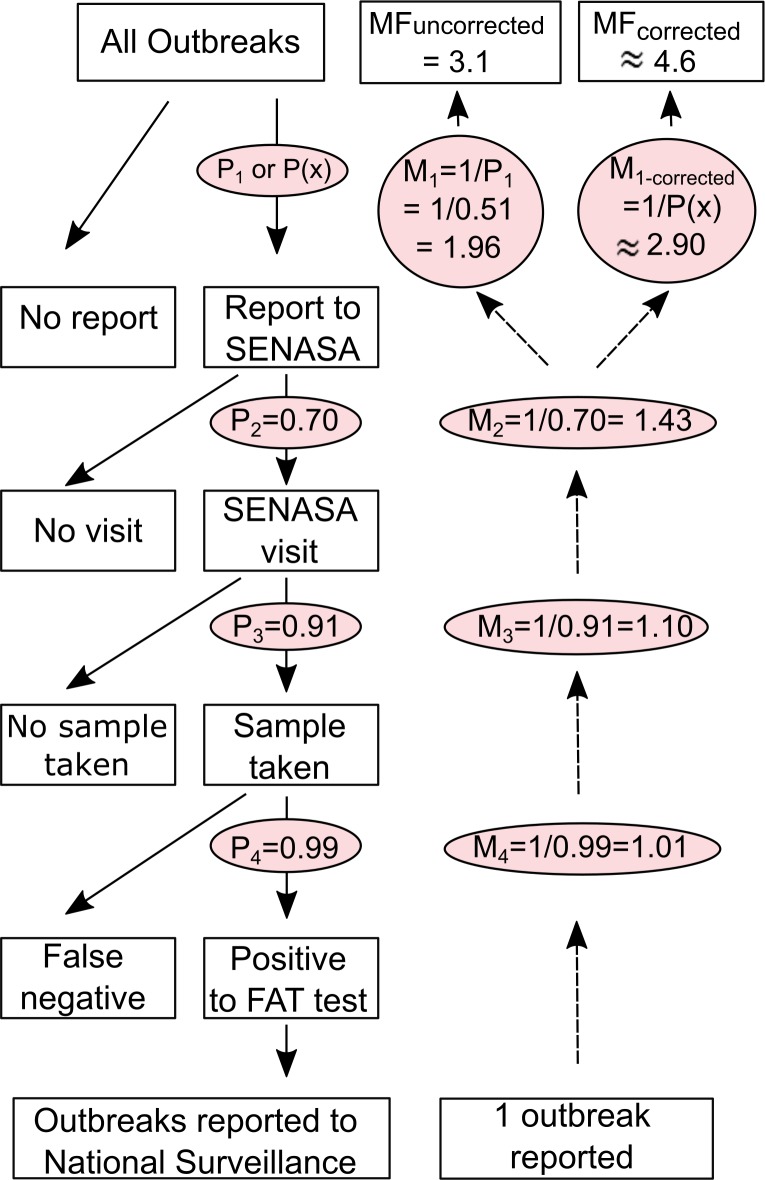
Calculation of multiplication factors from questionnaire and national surveillance data. Diagram illustrating the estimation of uncorrected (MF_uncorrected_) and spatially-corrected (MF_corrected_) under-reporting multiplication factors. (Left) Events occurring between an outbreak on a farm and its report and confirmation through the national surveillance system of Peru. The probability of each event is shown in parenthesis, based on results from our surveys, national surveillance records and the literature (for FAT test). (Right) The derived multiplication factors are calculated from these probabilities. For MF_corrected_, the spatial correction is applied to each outbreak by calculating probability p(x) of a farmer reporting a case, which is a function of its distance to the nearest reporting office. We show the value of the average MF_corrected_ based on confirmed outbreaks in 2014, with MF_corrected_ = $\sum_{i}^{N}\frac{1}{p(x_{i})}$, where N = total number of outbreaks and *p*(*x*_*i*_) = predicted reporting probability of outbreak *i* as a function of its distance *x*_*i*_ to the reporting office.

We used the method of Gibbons *et al*. [24] to estimate under-reporting of VBR cases in livestock and to calculate the multiplication factor (MF), defined as the multiplier needed to obtain an estimate of the actual number of VBR outbreaks from the number of reported outbreaks. We calculated the MF by multiplying the probabilities of four events that occur between the detection of a suspected outbreak and its entry into the national surveillance system (Fig 2). These four steps comprised the probability that a farmer reports a VBR outbreak (p1, estimated from questionnaires), the probability that SENASA attends a reported outbreak (p2, estimated from questionnaires), the probability that a sample was taken during the visit (p3, estimated from SENASA surveillance records) and the sensitivity of the fluorescent antibody test (FAT) for diagnosing VBR (p4). Although the FAT has high sensitivity (99%) under ideal laboratory conditions [38], sensitivity is lower in degraded samples that are transported from remote areas, resulting in false-negatives [39–41], and large variability has been observed in the performance of this test across laboratories of Latin America [42]. In our analysis, we assigned a constant probability to p4 for completeness of the framework (p4 = 0.99), but explored variation due to false negatives by calculating the MF separately for both laboratory-confirmed and for all suspected rabies outbreaks regardless of FAT results.

We estimated two different MFs that used either the average level of under-reporting across the area (MF_uncorrected_) or spatially explicit under-reporting rates that were corrected by the effect of geographic isolation from the nearest reporting office (MF_corrected_). The spatially explicit under-reporting rate was estimated from the glmmPQL model, where reporting probability was explained only by distance from the outbreak location to the office. The inverse of this probability was used to derive the ‘actual’ number of outbreaks. We assumed that no VBR outbreaks occurred in districts without a reported outbreak since 2003 (the year the surveillance system was implemented). Rabies absence from these putatively rabies-free areas was supported by statements from farmers that they had not observed clinical signs matching rabies in their animals (N = 0 out of 120).

We checked the consistency of our modelled burden estimates with a third approach that calculated the number of VBR outbreaks in livestock using farmers’ observations of clinical signs of rabies in their animals, rather than official surveillance data and claims of reporting tendencies from questionnaires. Specifically, we calculated this estimate (V) of cattle outbreaks from VBR as:

V=N×B×U×S Eq 1

where N is the total number of farms in districts with suspected outbreaks estimated from the 2012 National Census, B is the proportion of farms experiencing bat bites, U is the proportion of unvaccinated farms estimated from our surveys and S is the proportion of farmers from our survey that had observed specific clinical signs of rabies in their cattle during 2014. This estimate assumed that all cattle in a farm reporting vaccination are vaccinated. To correct this estimate by district-level differences, we estimated district specific parameters for each variable in Eq 1 from our surveys. For districts reporting outbreaks in 2014, but where no surveys were conducted (27/42 districts), we assigned the average estimate at the province- (23/27) or regional-level (4/27) for each variable.

The number of cattle deaths was calculated by multiplying the estimated number of outbreaks from each of these three methods (MF_uncorrected_, MF_corrected_, V) by the average number of cattle deaths per outbreak. The economic burden of VBR was then estimated by multiplying the actual number of cattle deaths by the average market value of a cow inferred from our surveys. Values of cows of different ages (adult, female and juveniles) were recorded in Peruvian New Soles (PNS) and converted to US$ using the average exchange rate of 2014 from the Central Bank Reserve of Peru (1US$ = 2.84 PNS).

Uncertainty in the estimation of each probability used to calculate the MFs and in the overall economic burden of VBR was modelled by resampling each probability from a binomial distribution with variance determined by the sample size of the data. The spatially-corrected under-reporting coefficient was resampled from a normal distribution determined by the model, while market prices and the number of cases per outbreak were sampled from their observed distributions. The uncertainty was assessed using 50,000 Monte Carlo simulations.

### Cost benefit analysis of cattle vaccination

The current cost of VBR to farmers includes both the expenses of vaccination and losses from livestock mortality. We compared relative costs of current practices (i.e., partial livestock vaccination and rabies mortality) to two alternative scenarios: vaccinating 100% of at-risk cattle (which would virtually eliminate VBR outbreaks) and forgoing vaccination entirely (which would presumably increase VBR outbreaks, but eliminate costs of vaccination). We used the cattle population reported in the 2012 census and the direct costs of vaccination per head of cattle to estimate cost scenarios. We assumed that under 100% coverage no livestock deaths would occur due to rabies, whereas under no vaccination, livestock deaths due to rabies would occur according to rates calculated in Eq 1, with U = 1. We used the estimated number of cattle deaths due to VBR from MF_corrected_ and the estimated number of unvaccinated cattle in the area to estimate a VBR incidence of unvaccinated cattle. Assuming a linear relationship between VBR incidence in our study area and cattle vaccination coverage, we then estimated the potential losses due to VBR mortality if no cattle were vaccinated as the total cattle population multiplied by the rabies incidence. We assume that cattle must be vaccinated annually for protection against VBR, as this is the current practice in Peru.

## Results

### Socio-ecological factors associated with disease reporting

**Fig 3:**
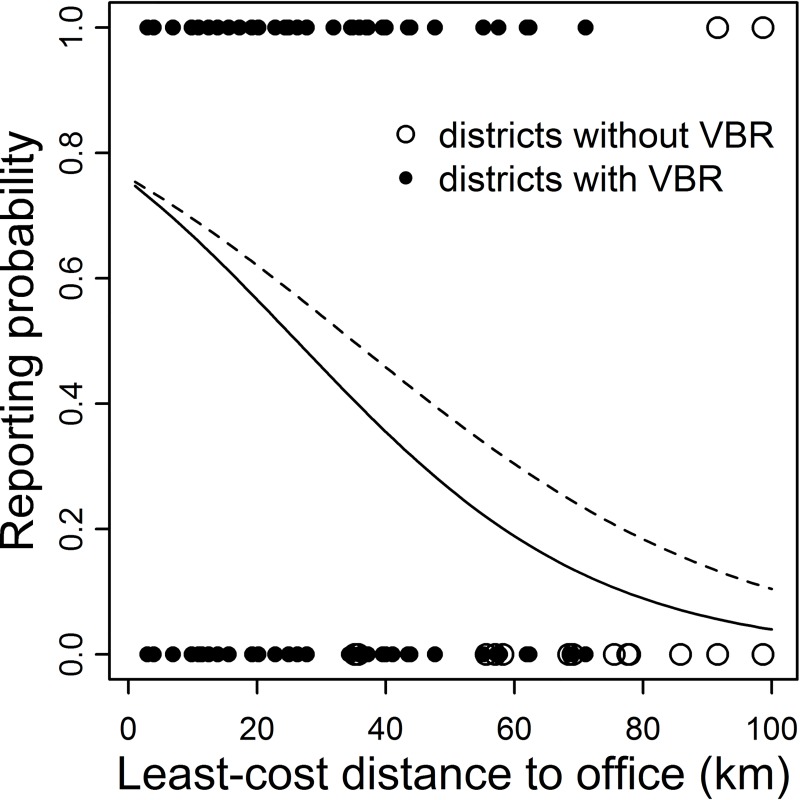
The effect of geographic isolation on the probability of reporting cattle deaths due to suspected infectious diseases. Dots show the responses of farmers in relation to reporting the mortality of a sick cow (1 = reporting, 0 = no reporting) as a function of their least-cost distance to the reporting office (estimated with the least-cost function, see methods). Farms were located either in districts with confirmed VBR outbreaks since 2013 (black dots) or in districts without any confirmed outbreaks (white dots). Only a single district (Chalhuanca) had suspected, but no confirmed rabies cases. Lines show the prediction of the glmmPQL model predicting reporting probability by the distance to the SENASA office using all districts (solid line) or only endemic districts (dashed line). The latest prediction was used to calculate a spatially-corrected under-reporting rate.

We interviewed 400 farmers between May and October 2015 in 40 communities (10 farmers per community) in 31 districts of 12 provinces in the Regions of Apurimac, Ayacucho and Cusco (Fig 1). The average number of cattle per farm was 10.6 (SD: 11.7, range: 1–151). On average, 38% of farmers stated they would report the death of a cow suspected to be caused by an infectious disease to SENASA. However, reporting varied from 0 to 100% across communities, with a lower tendency to report in districts that had not reported VBR from 2003 until the time of the study (6% vs 51% in districts with confirmed cases, Fig 1C). Reporting was spatially autocorrelated (Moran’s I test, p < 0.001) up to ca. 50 km, meaning farms located less than 50 km from each other had similar reporting patterns. Reporting declined with greater distances from reporting offices (Odds Ratio (OR) = 0.94, p < 0.01, Table 1, Fig 3). Reporting rates were more than twenty times lower in the Cusco region, where VBR arrived most recently, compared to Apurimac, which has a long history with rabies [15]. General perception of the risk of three diseases (VBR, Clostridiosis and FMD) increased the probability of reporting a dead cow by at least 80%, with the effect of VBR risk perception almost double the effect of risk perception of Clostridiosis or FMD (Table 1). Farms with larger herds were slightly less likely to report. Reporting was unrelated to farm elevation, two SES variables, respondent age or gender, knowing a veterinarian, or confidence in SENASA responding to a reported outbreak.

**Table 1:** Predictors of reporting cattle mortality due to suspected infectious disease in the southern Peruvian Andes.

Predictors	**Odds ratio (OR)**	**Value**	**Standard Error**	**t-value**	**p-value**
(Intercept)	-	0.634221	3.505103	0.18094	0.856
Distance to reporting office	0.94	-0.05952	0.015373	-3.8718	<0.001
Perception of rabies presence in community	3.11	1.133764	0.347386	3.2637	<0.01
Perception of Clostridiosis presence in community	1.84	0.60929	0.307971	1.9784	0.048
Perception of FMD presence in community	1.83	0.604164	0.305754	1.97598	0.049
Cusco Region	0.04	-3.11401	1.131002	-2.7533	<0.01
Ayacucho Region	0.69	-0.37782	0.856963	-0.4409	0.66
Number of cows	0.95	-0.0553	0.020709	-2.6703	<0.01
SENASA attends	1.76	0.56484	0.299631	1.88512	0.06
Gender (female)	0.59	-0.53441	0.295151	-1.8106	0.071
Socio Economic Status 1	0.88	-0.12475	0.106699	-1.1692	0.243
Socio Economic Status 2	1.09	0.086301	0.131578	0.65589	0.512
Age	1	-0.00485	0.011717	-0.4137	0.679
Knowledge of a veterinarian	0.62	-0.47994	0.319714	-1.5012	0.134
Elevation	1.00049	0.000492	0.001154	0.4264	0.67

### Socio-ecological factors associated with vaccinating livestock against rabies

Across the study area, 59% of farmers reported vaccinating their cattle against rabies. As with reporting, vaccination rates varied from 0–100% across communities (Fig 1D), and vaccination was spatially autocorrelated up to ca. 50km. Vaccination was generally performed by SENASA (78% of farmers that vaccinated), but 16% of farmers reported using a private or municipality veterinarian, and 5% of farmers vaccinated their animals themselves after purchasing vaccines from SENASA or private veterinarians. The vast majority (98%) of farmers paid the full cost of the vaccine and delivery (US$1.2 [SD: 0.3, range: 0.8–2.1]) from personal funds and 98% of those who vaccinated stated that they vaccinated all of their cows. Vaccination costs varied according to the price established by private veterinarians and the costs of the delivery and administration of vaccines. The main factor associated with vaccination was whether the farm was located in a district where a VBR case had been confirmed by SENASA prior to our surveys. Vaccination rates were 83% in farms located in districts with confirmed cases and 2% in farms located in districts without confirmed cases (Fig 1D). Thus, we tested factors associated with vaccination using a glmmPQL that included only data from farms in districts with confirmed outbreaks (N = 280). Vaccination against VBR was 13 times higher by respondents who also vaccinated against Clostridiosis, 3 times higher in farmers who stated they were aware of SENASA as an authority on animal health, and 7 times higher in farmers who lived in the Cusco region (Table 2). Vaccination slightly decreased at higher elevations (OR = 0.9960, p < 0.01). Neither distance to the SENASA office, the perceived risk of rabies in the community, socio-economic factors, perceived vaccine efficacy nor knowing a veterinarian were associated with vaccination (Table 2). Re-running the model replacing rabies risk perception by the last year that farmers perceived rabies in their community (modelled as a factor) showed that vaccination continued for the first 3 years after outbreaks, but decreased when rabies was perceived to be absent for 4 or more years (OR = 0.09, p = 0.02).

**Table 2:** Predictors of vaccinating cows against rabies in rabies-endemic districts.

Predictors	Odds ratio (OR)	Value	Standard Error	t-value	p-value
(Intercept)	-	10.2577	3.61658	2.81497	<0.01
Vaccination against Clostridiosis	13.45	2.59922	0.47153	5.32815	<0.001
Knowledge of SENASA	3.37	1.21503	0.588875	2.09879	0.04
Elevation	1	-0.0039	0.001182	-3.4786	<0.01
Cusco region	7.47	2.01074	1.033985	2.28448	0.063
Ayacucho region	0.37	-0.9955	0.775415	-1.721	0.211
Perception of rabies presence in community	2.26	0.81446	0.45876	1.77196	0.077
Distance to reporting office	1.02	0.01761	0.018297	1.76727	0.336
Socio Economic Status 1	1.02	0.01596	0.150919	0.16983	0.915
Socio Economic Status 2	0.87	-0.1378	0.187185	-0.6172	0.462
Age	0.99	-0.0063	0.018737	-0.329	0.736
Gender (female)	1.73	0.5499	0.442495	1.24783	0.215
Number of cows	1.03	0.02828	0.035162	0.70303	0.422
Knowledge of a veterinarian	0.51	-0.679	0.531072	-1.324	0.202
Perceived vaccine efficacy (medium)	1.16	0.14921	0.422253	0.30089	0.724

### VBR under-reporting multiplication factors

**Fig 4:**
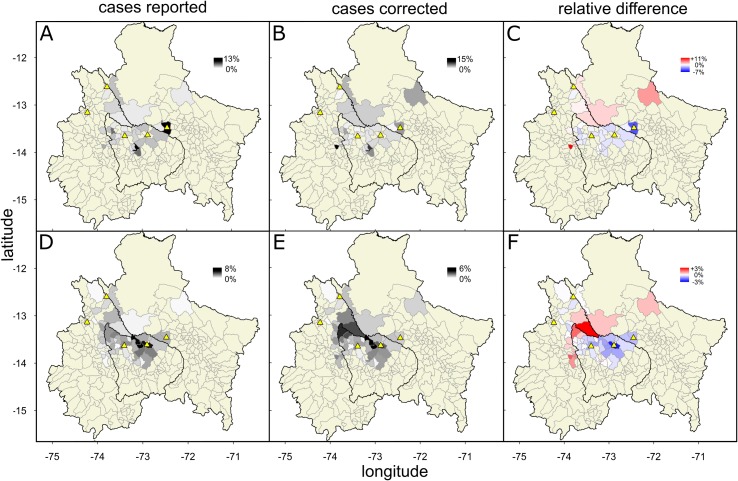
Spatial distribution of livestock rabies cases across districts in southern Peru after correcting for the effect of geographic isolation on reporting. (A) Relative percentage of cases per district across Ayacucho, Apurimac and Cusco regions that were reported to SENASA in 2014 (B) Relative percentage of cases per district in 2014 estimated after correcting by the effect of distance to the reporting office on reporting rates. (C) Relative difference between the official and the corrected number of cases in each district. (D) (E) and (F) are equivalent to (A) (B), (C) respectively for the period of 2003–2014. Districts coloured in beige do not have a VBR case reported and confirmed. Two cases from ‘Echarate’ district in Cusco were excluded from the 2003–2014 analysis given their very large distance to the reporting office, which generated a very high number of corrected cases and mislead the estimated relative proportion of cases of all other districts. Region and district maps were obtained from the GADM (http://www.gadm.org/) database using the *getData* function from the *raster* package of R.

Given that disease reporting by farmers was influenced by the local presence of VBR, we calculated the MF using only data from districts with at least one laboratory-confirmed VBR outbreak, which we assumed to reflect the actual presence of rabies in that district. This led to the exclusion of a single district (Chalhuanca) that had suspected, but no laboratory confirmed rabies cases. Without accounting for the effect of spatial isolation on reporting, we estimated an average MF_uncorrected_ of 3.1 (95% CI: 2.7–3.7) outbreaks for each reported outbreak (Fig 2). Incorporating the effect of distance to the office on reporting (OR = 0.970, p = 0.04, Fig 3) increased the MF_corrected_ to 4.6 (95% CI: 4.4–8.2). We also explored how this spatial correction of under-reporting affected the perceived distribution of outbreaks across the region either for the overall burden of VBR from 2003–2014 (assuming the effect of distance to office is constant across years), or for 2014 alone (Fig 4). This analysis highlighted districts in Ayacucho and Cusco that appeared to have relatively few outbreaks according to national surveillance records, but likely suffered a disproportionate number of outbreaks after adjusting for spatial effects on under-reporting. In contrast, districts near reporting offices in Apurimac had fewer outbreaks than implied by the raw data (Fig 4C and 4F).

### The burden of vampire bat rabies associated with cattle deaths during 2014

In 2014, 157 suspected outbreaks of VBR in cattle, associated with 169 deaths, were reported to SENASA in the regions of Ayacucho, Apurimac and Cusco, with 104 outbreaks (113 cases) laboratory-confirmed. The mean number of cattle deaths per outbreak was 1.06 (SD = 0.28, range 1–3), and the average price of cattle estimated from our surveys was mean ± SD: US$241 ± 134. For a total cattle population of 160,939 and 120,011 animals in districts with suspected and confirmed cases, this represents an official reported incidence of 105 suspected and 94 confirmed VBR deaths per 100,000 cattle. Across methods used to account for under-reporting, the estimated true number of VBR outbreaks during this period ranged from 341 (284 deaths/100,000 cattle) to 714 (444 deaths/100,000 cattle), representing economic losses of UD$81,524–171,992 (Table 3).

**Table 3:** Number of cases and economic burden of cattle losses due to VBR estimated by different methods for 2014 in southern Peru.

	Based on confirmed outbreaks		Based on suspected outbreaks	
Method	Number of cases (95% CI)	Economic loss in US$ (95% CI)	Number of cases (95% CI)	Economic loss in US$ (95% CI)
Official reports	113	26,974	169	26,974
MF_uncorrected_	341 (275–632)	81,524 (25,532–214,137)	468 (382–876)	112,934 (36,147–298,471)
MF_corrected_	505 (459–1165)	121,797 (47,009–408,013)	714 (680–1,629)	171,992 (66,973–573,879)
V	-	-	596 (354–1,042)	143,691 (40,742–371,897)
V_corrected_	-	-	522 (340–966)	125,813 (37,896–343,573)

The ‘MF_uncorrected_’ method estimated the actual number of cases using the average level of under-reporting across the region. The **‘**MF_corrected_’ estimated cases by correcting the under-reporting probability of each outbreak by the distance of that farm to the nearest reporting office. The ‘V’ method estimated cases multiplying the number of cattle by the percentage of cattle at risk and showing clinical signs of VBR as: V x number of cases per outbreak = 29389 × 0.76 × 0.18 × 0.14 × 1.06, with V _corrected_ estimating the number of cases based on district or province-level estimations.

At the national level in 2014, there were 254 suspected outbreaks, with 166 confirmed by FAT and 1.2 cases per outbreak (SD: 0.74, range: 1–10). Assuming a similar national MF_uncorrected_ to that estimated from our CBS, economic losses were estimated to UD$148, 841–206,840 (Table 4). Assuming the same level of under-reporting from 2003 to 2014, the economic burden of rabies had an average loss of US$150,876 (suspected cases) and US$ 93,554 (confirmed cases) per year.

**Table 4:** The number of cases and economic burden of cattle mortality from VBR in Peru during 2014.

	Based on confirmed outbreaks		Based on suspected outbreaks	
Method	Number of cases (95% CI)	Economic loss in US$ (95% CI)	Number of cases (95% CI)	Economic loss in US$ (95% CI)
Official reports	188	45,308	305	73,505
MF_uncorrected_	618 (443–1551)	148, 841(41,927–440,717)	859 (619–2151)	206,840 (58,712–608,596)

Estimates are given using the MF_uncorrected_ method.

### Cost benefit analysis of cattle vaccination

Vaccinating all cattle to eliminate the burden of VBR in districts with suspected cases would have cost US$194,496 in 2014, an average cost of US$12 per farmer. The vaccination coverage according to our surveys implies that farmers actually spent ~US$161,403 on cattle vaccination in 2014. Thus, the total cost of VBR (vaccination and MF_corrected_ rabies mortality) in 2014 was US$333,395. In the hypothetical scenario in which no cattle were vaccinated, our models project that 4,196 cattle would die of VBR annually (2,607 deaths/100,000 cattle), equivalent to an economic cost of US$1,010,560. Therefore, the current vaccination rate prevents approximately 3482 cattle deaths, a saving of ca. US$838,601. Under these assumptions, the benefit-cost ratio of vaccinating all cattle instead of no vaccination would be 5.2, and 1.71 compared to the current situation.

## Discussion

We identified factors associated with livestock disease reporting and vaccination against VBR, which we used to estimate the burden of VBR in southern Peru. After accounting for under-reporting, cattle VBR mortality was more than 4 times higher than the cost implied by official reports. At current vaccination levels, farmers in our study area spend approximately US$161,000 annually and still experience livestock losses due to VBR on the order of 444 deaths per 100,000 cattle. Together, animal mortality and vaccination costs exceed US$300,000 per year, representing a major loss for impoverished farming communities that rely on livestock for subsistence. Encouragingly however, our results suggest enhancing vaccination programs could dramatically diminish these financial losses.

Our estimates of economic costs of VBR cattle mortality in southern Peru in 2014 ranged from US$81,524 to US$171,992, depending on the method and on whether only laboratory confirmed or all suspected cases were considered. Given the lower sensitivity of the FAT test on degraded samples [40] and the significant reduction in reporting from farms located far from SENASA offices, we expect the true burden to be closer to our upper estimate. Costs towards this upper estimate were also supported by our independent calculation based on farmers’ personal observations of clinical signs of rabies in their animals (Table 3). The average monthly income in Ayacucho, Apurimac and Cusco for 2014 was US$243, and Ayacucho and Apurimac are among the poorest regions of the country [31]. Thus, the loss of a single cow from VBR (~US$241) is equivalent to approximately one month of income. Our surveys show that 61% of farmers used income from selling cows for household maintenance, and 30% for childhood education. Therefore these losses, while outwardly modest, may reinforce poverty among small-scale farmers in the Andean region that rely on livestock for subsistence, and consider livestock as ‘saving accounts’ [1, 43].

At the national level, cattle deaths from VBR costed US$148,742–206,840 during 2014. However, given the wide variation in reporting tendencies that we observed in our study region, similar studies in other areas are needed to further refine the total burden of VBR in Peru. For example, reporting could decrease more sharply with distance in areas where transportation is more limited, such as the Amazonian regions. Furthermore, regional differences in reporting and vaccination could occur independently of distance to the nearest reporting office, as we observed for Cusco. Nonetheless, our estimate can be used as a starting point when prioritizing efforts for disease control. For example, the national VBR burden is much lower than the economic losses estimated in Peru for parasites in llamas (~US$1.5 million [44]) but equivalent to the burden of Echinococcis (~US$196,000 only for direct losses [45]).

It is important to acknowledge additional costs of VBR that we were unable to include. First, we did not directly quantify the losses associated with dairy production, which was practiced by 60% of farmers in our study. However, we expect that the price of a cow will account for part of this cost. Second, although almost 90% of reported VBR outbreaks in Peruvian livestock involve cattle [15], even greater under-reporting of less valued livestock species (e.g., goats, pigs) is likely [46]. Third, the average number of deaths per outbreak was reported in the surveillance system as the number of dead or sick animals during the SENASA visit, but additional animals that died after the visit would not have been included in our estimates. We also excluded data from districts with no official reports of rabies, which would make our estimates overly conservative if rabies were actually present. However, questionnaires confirmed the absence of animals with clinical signs of rabies in putative rabies-free districts (compared to 14% in endemic districts), and our previous epidemiological analyses of travelling waves of VBR implied that these areas are truly rabies-free [15]. Thus, while we expect the bias introduced by this assumption is minimal, delayed reporting in newly infected might still occur which would increase the estimated number of outbreaks to higher than reported here [47]. Finally, data were unavailable to estimate the financial costs of sampling and diagnostic testing of suspected rabies cases. All together, these factors are likely to increase the net cost of VBR beyond the estimates presented in this study.

We also demonstrated a statistical framework to incorporate spatial heterogeneity in reporting practices into estimates of disease burden. Although we corrected the estimated burden by using only distance to reporting offices, in principle our approach can be generalized to include other factors affecting reporting or diagnostic sensitivity when these are available. In our dataset, correcting for spatially heterogeneous under-reporting revealed geographic areas that had a disproportionately higher burden of VBR than implied by official records (Fig 4). This finding highlights the possibility that resources for prevention and control will be directed to areas that have high reporting, but not necessarily the highest burden, which could amplify disparities in VBR burden. Moreover, given that VBR persists in bats through spatial dynamics, neglected high burden/low reporting areas could create hotspots of transmission that facilitate long term viral persistence [48].

Our results support the findings from previous studies that livestock vaccination is the most effective intervention to reduce the burden of VBR [11, 17]. In our study, vaccination coverage against VBR was high (83%) in districts with confirmed outbreaks, but almost non-existent (i.e. 2% of farmers) in neighbouring, putatively rabies-free districts [15]. This shows that vaccination occurs reactively to VBR outbreaks and provides a mechanism (lower vaccination rates and reduced herd immunity) by which outbreaks in newly invaded areas might be larger than in historically endemic areas. Within VBR endemic areas, distance to the reporting office was not correlated with vaccination, suggesting that the presence of VBR outweighs logistical challenges to acquire vaccines. Given the high cost of this vaccine to farmers (around US$1.2 per dose), it was surprising that socio-economic factors were unrelated to vaccination, suggesting that the perceived risk of rabies is more important than affordability in driving vaccination uptake for VBR.

We estimated that the current vaccination coverage of 83% prevented the death of around 3842 cattle in 2014, which saved farmers ~US$800,000. These savings should be treated with caution since farmers may have over-stated vaccination rates and independent confirmation (e.g., vaccination certificates) were unavailable. Moreover, our estimate assumes a linear relationship between vaccination coverage and VBR incidence, which while intuitive, has not been empirically demonstrated, and reactive vaccination in response to outbreaks may further complicate this relationship. Nonetheless, our results imply that vaccinating all cattle would be 1.7 times more beneficial than the current vaccination coverage, and 5 times more beneficial than not vaccinating cattle due to the expected increase in livestock mortality. The latter ratio is similar to the benefit-cost ratio of 6 estimated in Mexico [17]. Therefore, our results suggest that further investments in cattle vaccination, perhaps through government subsidies, would be economically beneficial to mitigate the burden of VBR. However, vaccinating the remaining cattle population could be more challenging and costly than achieving the current coverage, especially if the remaining cattle population is owned by farmers that are reluctant to vaccinate because they do not perceive VBR as a threat.

To our knowledge, our study is the first estimate of the burden of VBR in Latin America to incorporate estimated under-reporting rates or spatial heterogeneity in reporting and disease occurrence. This estimate, at least four times higher than official reports, is essential in planning and implementing cost-effective measures to prevent and control the disease, which mainly affects low-income, small-scale farmers. Our results further suggest that increasing the risk perception of communities that are far from reporting offices could both increase reporting and reduce cattle losses by encouraging preventative vaccination in high risk areas. This could be achieved by developing awareness campaigns using relatively inexpensive tools like community radios. More broadly, this work highlights how variation in disease reporting can influence estimates of disease burden, which will be important to consider when extrapolating burden estimates from community-based studies across larger spatial scales.

## Supporting information

S1 Table Farmer answers from surveys. This table includes farmers’ responses and additional information analysed in this study.
(XLSX)

S2 Table Questions from the survey and type of variable associated for the regression analysis or the burden estimation. (XLSX)
